# Supplementary material for: Isolation of Haustorium Protoplasts Optimized by Orthogonal Design for Transient Gene Expression in Phelipanche aegyptiaca
Source: Plants (Basel). 2024 Aug 5;13(15):2163. doi: 10.3390/plants13152163 (PMC11314320; doi:10.3390/plants13152163)
Supplement: Supplementary file 1 [file plants-13-02163-s001.zip › plants-3115097-supplementary.pdf]

**Table S1.** The yield and activity of protoplasts under six-factor and five-level orthogonal experiment

| Treatment No. | Cellulase R-10 (%) | Macerozyme R-10 (%) | Mannitol (mol/l) | enzymolysis time (h) | enzymolysis temperature (°C) | centrifugal gravity (×g) | Protoplast yield (×$10^6$ protoplasts·g $FW^{-1}$) | Protoplast viability (%) |
|---|---|---|---|---|---|---|---|---|
| 1 | 1.5 | 0.6 | 0.3 | 2 | 23 | 60 | 2.33 | 84.31 |
| 2 | 1.5 | 0.7 | 0.6 | 6 | 24 | 90 | 5.82 | 79.89 |
| 3 | 1.5 | 0.8 | 0.4 | 5 | 25 | 70 | 3.25 | 89.62 |
| 4 | 1.5 | 0.9 | 0.7 | 4 | 26 | 100 | 4.38 | 73.33 |
| 5 | 1.5 | 1 | 0.5 | 3 | 27 | 80 | 4.67 | 78.73 |
| 6 | 2 | 0.6 | 0.5 | 5 | 26 | 90 | 4.20 | 87.90 |
| 7 | 2 | 0.7 | 0.3 | 4 | 27 | 70 | 3.95 | 78.98 |
| 8 | 2 | 0.8 | 0.6 | 3 | 23 | 100 | 5.75 | 74.20 |
| 9 | 2 | 0.9 | 0.4 | 2 | 24 | 80 | 3.32 | 80.43 |
| 10 | 2 | 1 | 0.7 | 6 | 25 | 60 | 3.62 | 69.16 |
| 11 | 2.5 | 0.6 | 0.7 | 3 | 24 | 70 | 4.50 | 74.39 |
| 12 | 2.5 | 0.7 | 0.5 | 2 | 25 | 100 | 3.80 | 87.12 |
| 13 | 2.5 | 0.8 | 0.3 | 6 | 26 | 80 | 5.03 | 79.04 |
| 14 | 2.5 | 0.9 | 0.6 | 5 | 27 | 60 | 4.28 | 76.11 |
| 15 | 2.5 | 1 | 0.4 | 4 | 23 | 90 | 6.74 | 76.59 |
| 16 | 3 | 0.6 | 0.4 | 6 | 27 | 100 | 4.71 | 84.78 |
| 17 | 3 | 0.7 | 0.7 | 5 | 23 | 80 | 4.45 | 70.59 |
| 18 | 3 | 0.8 | 0.5 | 4 | 24 | 60 | 7.41 | 82.12 |
| 19 | 3 | 0.9 | 0.3 | 3 | 25 | 90 | 5.25 | 71.61 |
| 20 | 3 | 1 | 0.6 | 2 | 26 | 70 | 3.58 | 66.48 |
| 21 | 3.5 | 0.6 | 0.6 | 4 | 25 | 80 | 7.12 | 74.61 |
| 22 | 3.5 | 0.7 | 0.4 | 3 | 26 | 60 | 5.92 | 82.59 |
| 23 | 3.5 | 0.8 | 0.7 | 2 | 27 | 90 | 3.15 | 72.39 |
| 24 | 3.5 | 0.9 | 0.5 | 6 | 23 | 70 | 6.84 | 78.90 |
| 25 | 3.5 | 1 | 0.3 | 5 | 24 | 100 | 8.51 | 63.07 |
